# Transcriptional Dynamics and Candidate Genes Involved in Pod Maturation of Common Bean (*Phaseolus vulgaris* L.)

**DOI:** 10.3390/plants9040545

**Published:** 2020-04-22

**Authors:** Cristina Gómez-Martín, Carmen Capel, Ana M. González, Ricardo Lebrón, Fernando J. Yuste-Lisbona, Michael Hackenberg, José L. Oliver, Marta Santalla, Rafael Lozano

**Affiliations:** 1 Departamento de Genética, Facultad de Ciencias & Laboratorio de Bioinformática, Centro de Investigación Biomédica, Universidad de Granada. 18071 Granada, Spain; cris12gm@gmail.com (C.G.-M.); hackenberg@go.ugr.es (M.H.); oliver@ugr.es (J.L.O.); 2 Centro de Investigación en Biotecnología Agroalimentaria (BITAL), Universidad de Almería. 04120 Almería, Spain; ccapel@ual.es (C.C.); fyuste@ual.es (F.J.Y.-L.); 3 Grupo de Genética del Desarrollo de Plantas, Misión Biológica de Galicia – CSIC. P.O. Box 28. 36080 Pontevedra, Spain; amgonzalez@mbg.csic.es (A.M.G.); msantalla@mbg.csic.es (M.S.)

**Keywords:** pod maturation, transcription factors, photosynthesis, metabolism, *Phaseolus vulgaris*

## Abstract

Pod maturation of common bean relies upon complex gene expression changes, which in turn are crucial for seed formation and dispersal. Hence, dissecting the transcriptional regulation of pod maturation would be of great significance for breeding programs. In this study, a comprehensive characterization of expression changes has been performed in two common bean cultivars (ancient and modern) by analyzing the transcriptomes of five developmental pod stages, from fruit setting to maturation. RNA-seq analysis allowed for the identification of key genes shared by both accessions, which in turn were homologous to known Arabidopsis maturation genes and furthermore showed a similar expression pattern along the maturation process. Gene- expression changes suggested a role in promoting an accelerated breakdown of photosynthetic and ribosomal machinery associated with chlorophyll degradation and early activation of alpha-linolenic acid metabolism. A further study of transcription factors and their DNA binding sites revealed three candidate genes whose functions may play a dominant role in regulating pod maturation. Altogether, this research identifies the first maturation gene set reported in common bean so far and contributes to a better understanding of the dynamic mechanisms of pod maturation, providing potentially useful information for genomic-assisted breeding of common bean yield and pod quality attributes.

## 1. Introduction

Angiosperms have evolved to produce fruits that allow for optimal seed development in a closed protective structure with multiple shapes and sizes, which facilitates seed development and dispersal. Studies on the Arabidopsis and tomato model species have significantly advanced the current understanding of molecular and genetic basis that control dry and fleshy fruit development, respectively [1,2,3,4,5,6]. Thus, despite the differences between dry and fleshy fruit developmental patterns, strong similarities have been found in the molecular circuits governing their development and maturation programs, indicating that fruit regulatory networks are conserved across a broad spectrum of angiosperms [1,2]. Among these conserved physiological and metabolic processes, the decay on photosynthesis and translation ribosome machinery led to the deactivation of green stage-related processes and the production of secondary color metabolites such as carotenoids and anthocyanins [7,8,9,10,11]. Likewise, jasmonate promotes senescence and plays an important role in chlorophyll degradation [12,13], which is synthesized from alpha-linolenic acid [14,15], suggesting that the accumulation of this acid during maturation is responsible for stimulating chlorophyll loss [16]. In addition, the increased accumulation of antioxidant and monoterpene is also an important feature of the fruit maturation process [17,18,19].

Like most species of the *Fabaceae* (also named as *Leguminosae*) family, common bean (*Phaseolus vulgaris* L.) produces dry fruits in the form of a pod, which is a photosynthetically active organ that encloses the developing seeds and protects them from pests and pathogens. Indeed, photosynthetically active pod tissue contributes to fuel seed growth with assimilates and nutrients [20]. As in most angiosperms, significant genetic, biochemical, and physiological changes take place during fruit maturation, which confers the functional and morphological properties of this plant organ. Unlike members of the *Brassicaceae* family that produce pods (siliques) derived from two fused carpels, legume pods are developed from a single carpel that emerges from the gynoecium after ovule fertilization. Subsequently, pods attain their maximum size through a growth phase caused by an active cell division and later cell expansion [21]. During the maturation phase, carbohydrate metabolism and amino acid biosynthesis are increased in pods, leading to the accumulation of storage compounds, mainly starch, total soluble amino acids, and sugars [22,23]. In later stages of the maturation phase, the green pod stage, which coincides with the first two phases of seed development, gives way to the chlorophyll-loss stage where pods acquire yellow hues.

The common bean is the most commonly consumed legume worldwide for its edible dry seeds and pods. Originating in the New World and widespread because of its broad adaptation, evidence points towards the domestication in the Andes and Mesoamerica, independently [24,25]. Within each of these two major domesticated gene pools, a wide range of fruit morphologies adds to the variation in developmental rates [26,27]. Thus, common bean pods are highly variable and range from tiny single-seeded forms to many-seeded fruits, from fruits with reduced wall fiber, absence of suture strings (stringless), succulent walls, and completely indehiscent (for example snap bean varieties) to dehiscent, with the presence of fibers in both their sutures (strings) and walls [28,29,30]. The synchronous growth and development of the fruit and the seed structures are essential for the formation of viable seeds and of interest in breeding programs for common bean. However, genetic and molecular studies on the mechanisms regulating fruit development and maturation are scarce. Therefore, the precise identification of gene expression changes and pathway signatures underlying the coordinated development of pericarp and seeds would be critical to envisage how to reduce seed yield losses in the field and increase edible pod quality in common bean, two important objectives of the current breeding programs of this crop species.

In order to reveal differentially expressed genes (DEGs) between pre- and post-maturation phases of common bean, a high-through-output RNA-seq analysis [31] was performed in five pod developmental stages of this species. The analyses were replicated in two accessions contrasting in several developmental and agronomical traits, with the aim to uncover those DEGs shared by both accessions, thus allowing for the identification of genes involved in common bean maturation process regardless of the accession type. The subsequent functional enrichment in GO terms and KEGG pathways, as well as its comparison to homologous Arabidopsis maturation genes, made it possible to refine this list and identify key specific genes involved in common bean pod maturation. This study’s results may be a valuable source of information for pod and seed production improvement in common bean, which will also be used for the breeding of other legume species.

## 2. Results

### 2.1. Pod Development of PMB0225 and PHA1037 Common Bean Accessions

**Figure 1:**
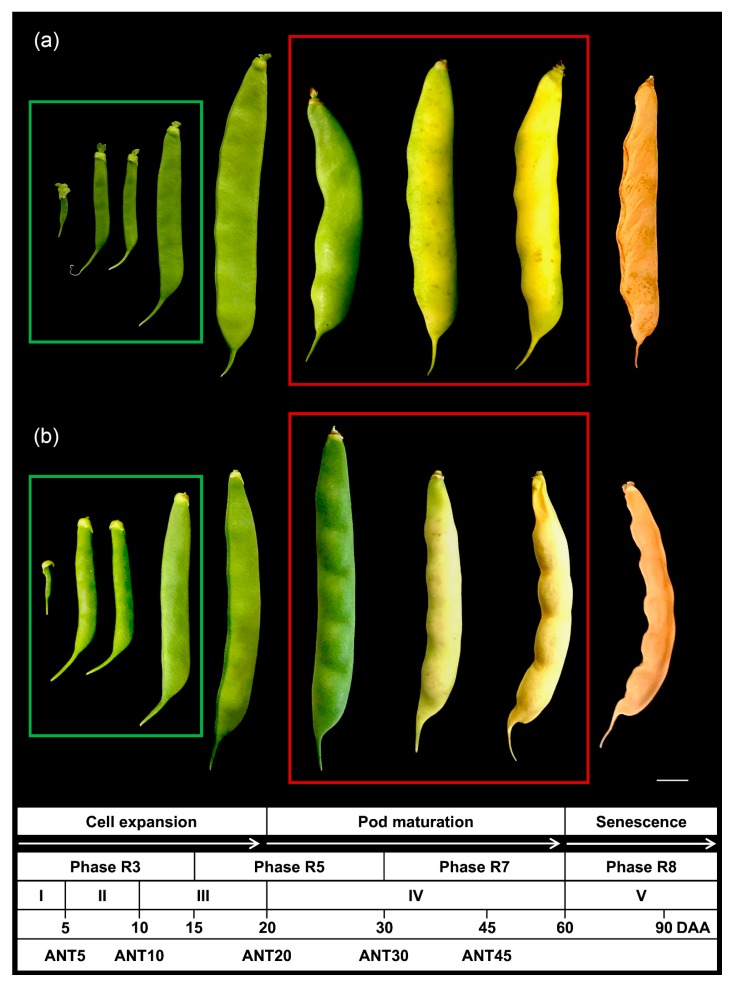
Growth characteristics of pods harvested at different time points after anthesis (DAA) during development of the cultivated breeding line PMB0225 (**a**) and the weedy-form PHA1037 (**b**). The key phases and stages of pod development are assigned according to [32,33] as: R3 = first pod larger than 2.5 cm; R5 = beginning of rapid seed growth; R7 = physiological maturity; and R8 = harvest maturity; Stages I (0−5 DAA) = initial exponential growth; II (5−10 DAA) = linear growth; III (10−20 DAA) = cessation of pod elongation; IV (20−60 DAA) = maturation; and V (60−90 DAA) = senescence. Additionally, taking as reference the day of anthesis (ANT), sub-stages of pod development used for transcriptomic analysis were labeled as ANT5, ANT10, ANT20, ANT30, and ANT45. ANT5 and ANT10 were considered as immature stages (green rectangle), whereas ANT20, ANT30, and ANT45 were considered as mature stages (red rectangle). Scale bar = 1 cm.

So as to gain insights into the pod development and maturation of common bean, two contrasting accessions were evaluated, the PMB0225 cultivated breeding line and the weedy-form PHA1037 nuña bean. PMB0225 is a long-day photoperiod-adapted line with indehiscent and stringless fruits, whereas PHA1037 is a long-day photoperiod-sensitive line that yields dehiscent and stringy pods. Based on growth characteristics, the common bean reproductive development can be divided into four major phases (R3, R5, R7, and R8) and five key stages (from I to V) that comprise developmental processes from flower anthesis to pod senescence [28,32,33]. Under short-day photoperiod growth conditions, both accessions took approximately 90 days after anthesis (DAA) to successfully complete their reproductive development (Figure 1).

Upon ovule fertilization and during the R3 phase, developing pods grew exponentially until five DAA (Stage I) and the first pod reached a length higher than 2.5 cm. The pericarp and seeds developed as one physiological unit at this stage. Later, pod development was characterized by a linear growth rate where seeds started to differentiate at Stage II. Pods elongated rapidly until 15 DAA, which corresponds to the end of the R3 phase. At the beginning of the R5 phase, there was rapid seed growth and cessation of pod elongation (Stage III). The onset of maturation was reached at approximately 20 DAA; since then, seeds underwent further development and pods started to turn yellow (Stage IV). The end of the rapid seed growth marked the R7 phase at 30–60 DAA during which pods achieved physiological maturity. At the R8 phase, pod senescence was initiated and gave rise to seed and pod desiccation during a harvest maturity stage (Stage V). In addition, for a better understanding of the transcriptomic comparative analysis reported in this work, several sub-stages of pod development have been considered, taking as reference the day of anthesis (ANT). Thus, uniformly sized pods were collected at 5, 10, 20, 30, and 45 DAA at developmental sub-stages ANT5 (R3-I), ANT10 (R3-II), ANT20 (R5-III), ANT30 (R5-IV), and ANT45 (R7-IV), as indicated in the Materials and Methods section.

### 2.2. Gene Expression Changes Occurring during Pod Maturation of Common Bean

**Figure 2:**
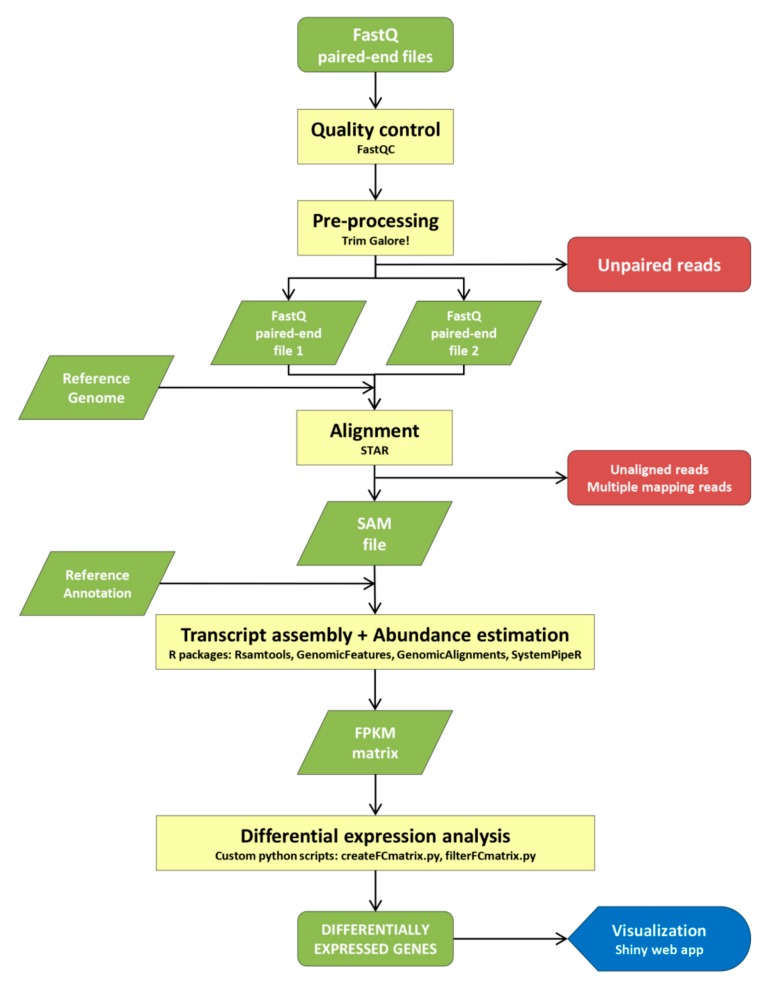
RNA-seq bioinformatics protocol used to identify differentially expressed genes in common bean.

To unravel the transcriptomic changes associated with the different developmental stages of pod maturation in the crop legume common bean, an RNA-seq bioinformatics approach (Figure 2) was developed to compare immature to mature stages of pod development in the two contrasting accessions (PMB0225 and PHA1037). The purpose of using contrasting accessions was to discard genotype-specific maturation genes, and to identify those DEGs involved in the maturation process of common bean independently of the genotype’s origin. Thus, a total of three biological replicates were analyzed for each genotype and developmental stage. The consistency of RNA-seq biological replicates was determined by hierarchical clustering analysis of the top-ranked 100 expressed genes, which were common across all samples (see Materials and Methods for details). As a result, the replicates R2 of ANT30 and ANT45 from PMB0225, and the replicate R2 of ANT45 from PHA1037 were rejected for further analyses as they showed a clustering behavior discordant to the other two respective biological replicates (Figure S1, Table S1).

**Figure 3:**
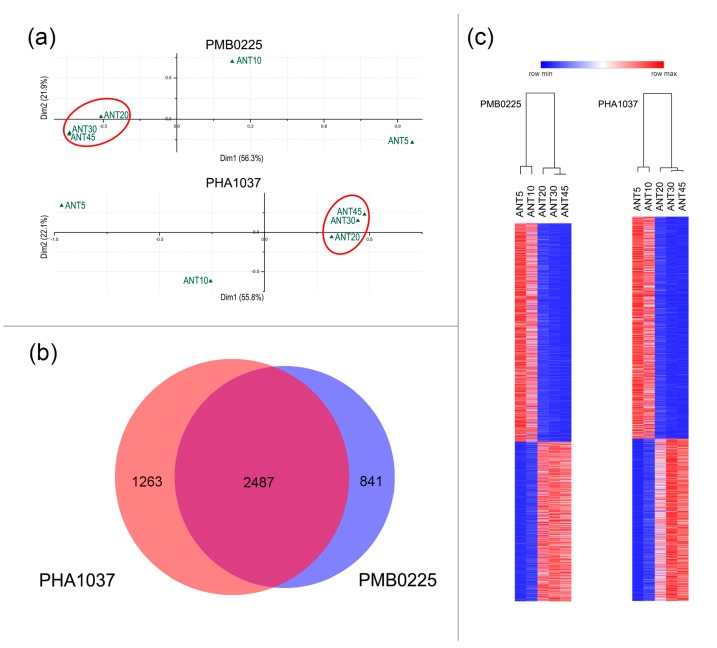
Genes differentially expressed between mature (ANT20 + ANT30 + ANT45) and immature (ANT5 + ANT10) pod stages. (**a**) Correspondence multivariate analyses based on all expressed genes in PMB0225 and PHA1037 along the different fruit developmental stages ANT5, ANT10, ANT20, ANT30, and ANT45. The variances absorbed by the two first factors (Dim1 and Dim2) at each plot are indicated in parentheses. Mature stages (ANT20, ANT30, and ANT45) are enclosed in red ovals. (**b**) Venn diagram of differentially expressed genes in PMB0225 and PHA1037 accessions during fruit development and maturation stages. The 2487 genes shared by both cultivars can be considered as candidate maturation genes. In addition, other 841 DE genes were exclusively found in PMB0225 and 1263 in PHA1037. (**c**) Hierarchical clustering of 2487 candidate maturation genes in PMB0255 and PHA1037 cultivars. The clusters of up- and down-regulated genes at maturation stages (ANT20, ANT30, and ANT45), as compared to the immature ones (ANT5 and ANT10), are clearly shown.

Using the matrix of fragments per kilobase of transcript per million (FPKM) values for the 27,433 annotated genes in the five developmental stages of each accession; global gene expression variation was first explored by applying a multivariate correspondence analysis [34,35]. As shown in Figure 3a, Factor 1, explaining above 50% of total variance, clearly separates ANT5 and ANT10 immature stages from the mature ones ANT20, ANT30, and ANT45, in both cultivars.

With the aim to identify genes differentially expressed between immature and mature pod stages, the fold change (FC) in gene expression between the different developmental stages was computed in FPKM values. Using custom python scripts [36], FC values higher than two or lower than −2, i.e., |FC|>2, were considered significant. Those genes with a significant FC in mature stages as compared to immature stages were considered as DEGs. Thus, 3328 DEGs were identified in the PMB0225 breeding line and 3750 in PHA1037 weedy accession. From these, 2487 DEGs are shared by both accessions and their expression profiles and hierarchical clustering analysis clearly indicated that 1050 were up-regulated and 1437 were down-regulated at maturation stages (Figure 3b,c). Therefore, these 2487 DEGs were tentatively regarded as candidate genes involved in common bean pod maturation. The FPKM values of these candidate maturation genes in the five studied developmental stages of each accession are shown in Tables S2.1 and S2.2, and can be visualized in our web server [37].

**Figure 4:**
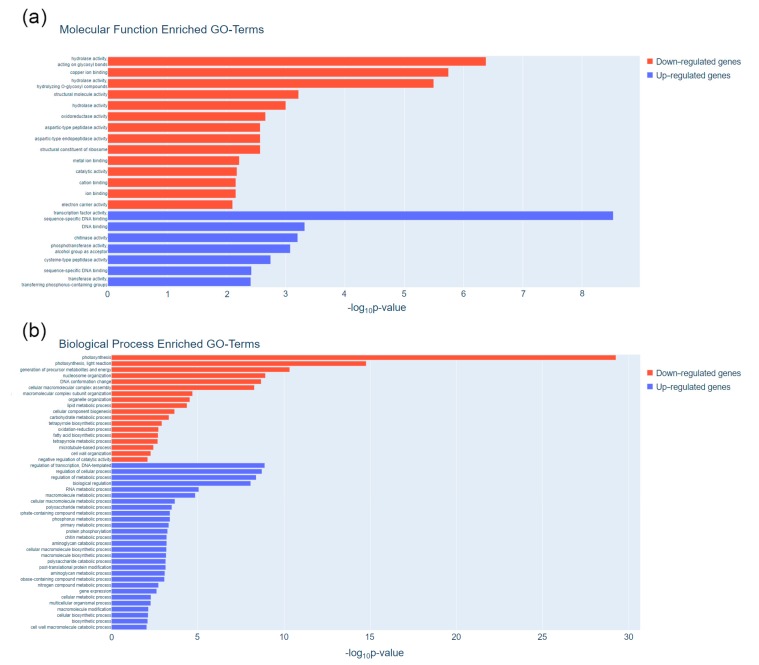
Curated list of significantly enriched GO terms for (**a**) Molecular Function and (**b**) Biological Process categories in down-regulated (red) and up-regulated (blue) candidate maturation genes. Significance scores were calculated as the -log10 of the *p*-value. The full list of significantly enriched GO terms is available in Tables S3.1 and S3.2.

The functional relevance of candidate pod maturation genes was determined by performing GO-term enrichment analysis. The scatterplots for biological process and molecular function ontologies enriched in the candidate maturation genes are shown in Figure 4. Among up-regulated genes, the most significant biological process enriched terms were regulation of both cellular process and transcription as well as the metabolism of RNA and other biological molecules, whereas the most significantly enriched terms for molecular function were transcription factor activity and transcription regulator activity (Figure 4a,b, Table S3.1). Within the group of down-regulated genes, photosynthesis, nucleosome organization, and lipid metabolism were the most statistically significant terms for biological processes. Hydrolase activity, acting on glycosyl bonds, hydrolase activity, hydrolyzing O-glycosyl compounds, and copper ion binding were the most significant molecular function enriched terms (Figure 4a,b, Table S3.2).

**Figure 5:**
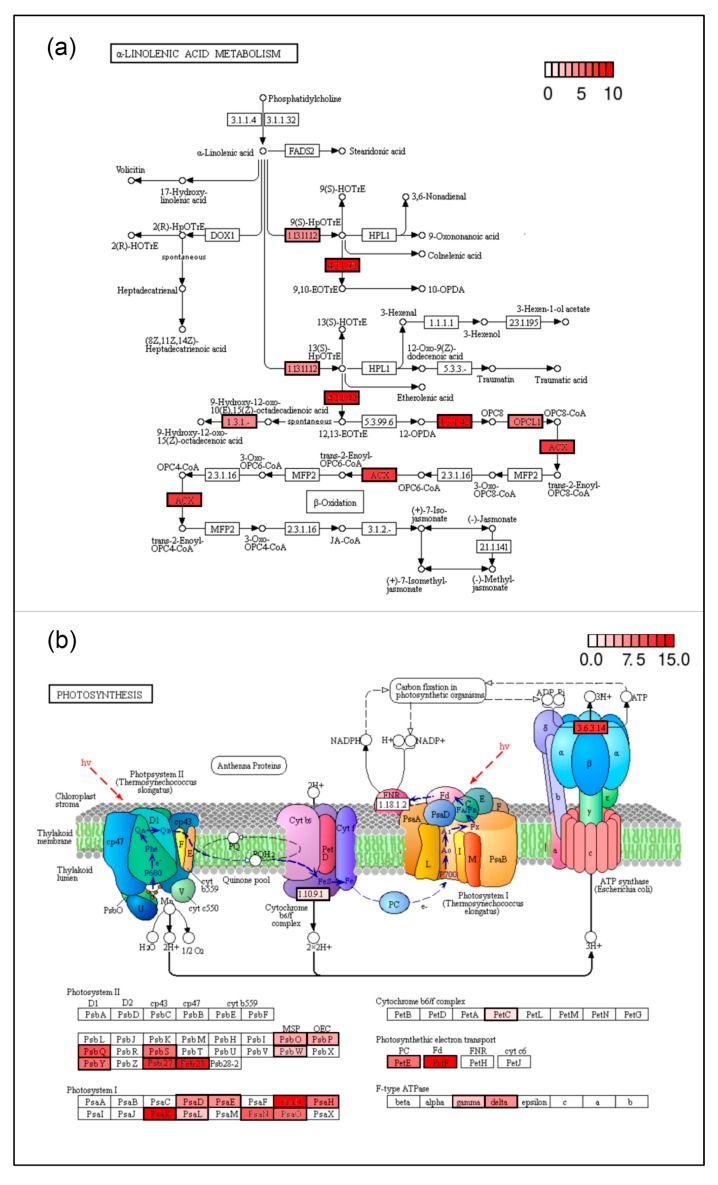
KEGG pathways highlighting the differentially expressed genes identified in this study. (**a**) Up-regulated maturation genes in the α-linolenic acid metabolism KEGG pathway (pvu00592). (**b**) Down-regulated maturation genes in the photosynthesis KEGG pathway (pvu00195). The scale indicates the average |FC| values between immature (ANT5 and ANT10) and mature (ANT20, ANT30, and ANT45) stages. Gene acronyms are used in this figure to designate the respective *Phaseolus* genes (see Table S4).

To further characterize the pod maturation process, the enrichment of up- and down-regulated candidate maturation genes in KEGG pathways [38] was analyzed. Then, the Pathview package [39] was used to map and visualize the candidate maturation genes involved in the significantly enriched KEGG pathways. Thus, 11, 10, 20, and 19 up-regulated maturation genes were found to be associated with the enriched pathways (adjusted *p*-value ≤ 0.01) related to glutathione metabolism, alpha-linolenic acid metabolism (Figure 5a), MAPK signaling pathway and plant–pathogen interaction, respectively (Table S4.1). On the other hand, 30, 22, and 16 down-regulated maturation genes were involved in the photosynthesis (Figure 5b), photosynthesis–antenna proteins, porphyrin, and chlorophyll metabolism enriched pathways (Table S4.2). Furthermore, fatty acid biosynthesis and ribosome pathways were also analyzed due to their relevance in the maturation process according to the recent bibliography [5,40], in which 7 and 30 genes were identified, respectively (Table S4.2).

### 2.3. Comparative Analysis of Pod Maturation Process in Common Bean and Arabidopsis

In order to investigate the degree of analogy of the fruit maturation process between common bean and the *Arabidopsis thaliana* model species, candidate pod maturation DEGs here identified were compared with those reported by Mizzotti et al. [41] in Arabidopsis. As a result, 189 up-regulated homologous maturation genes were found to be shared between both plant species (Table S5.1). Among them, genes encoding transcription factors of the NAC family such as the homologous to Arabidopsis *ATAF1* (Phvul.009G125900), *NAC2* (Phvul.005G084600, Phvul.011G148000), *NAC3* (Phvul.005G084500), *NAC14* (Phvul.009G039000), *NAC46* (Phvul.001G100500), and *NAC100* (Phvul.003G189000, Phvul.009G186000). Several of these NAC transcription factors control the expression of chlorophyll catabolic and senescence-associated genes [42,43], and may act as positive regulators of the pod maturation program. Thus, in the legume crop soybean (*Glycine max* (L.) Merr.), a reduction in maturation-associated pod dehiscence is caused by a regulatory mutation in the SHAT1-5 NAC transcription factor [44]. Similarly, genes encoding GRAS transcription factors, like Phvul.003G085000 and Phvul.006G141700, homologous to the Arabidopsis *SCARECROW*-*LIKE13* [45] and WRKY transcription factors like *WRKY48* (Phvul.003G116300), *WRKY69* (Phvul.005G093800), and *WRKY75* (Phvul.001G088200) genes, showed an induced expression pattern both in Arabidopsis and common bean mature pods. Remarkably, these latter genes have a functional role in the genetic network regulating ripening-associated genes, particularly those involved in color changes and chlorophyll degradation in tomato fruits [46].

In addition, 521 homologous maturation-related genes showing a down-regulated expression pattern during pod development were found to be shared between common bean and Arabidopsis (Table S5.2). This set of genes included expansin genes such as *EXPA3* (Phvul.010G010200, Phvul.011G063800), *EXPA6* (Phvul.008G232200), *EXPA8* (Phvul.006G077200), *EXPA11* (Phvul.009G019000), and *EXPB3* (Phvul.005G104600, Phvul.011G117500). In addition to cell wall polysaccharides, the expansins may be involved in cell enlargement events by breaking the H-bonds between hemicellulose and cellulose and allowing shear of the cellulose fibrils [32]. Likewise, three fasciclin-like arabinogalactan protein genes homologous to the Arabidopsis *FLA2* (Phvul.008G288800), *FLA7* (Phvul.008G287700), and *FLA10* (Phvul.008G075000) were down-regulated during maturation. Interestingly, several FLA proteins have been involved in modulating the biosynthesis of cell wall polysaccharides during the formation of cotton fiber [47].

KEGG pathways enrichment analysis of the fruit maturation genes shared between these two species was also performed (Table S6). It was found that there were no KEGG-enriched pathways in up-regulated maturation genes; however, down-regulated genes were enriched in photosynthesis, photosynthesis—antenna proteins, porphyrin and chlorophyll metabolism, and ribosome pathways.

### 2.4. Identification of Putative Transcriptional Regulators Involved in Pod Maturation

**Figure 6:**
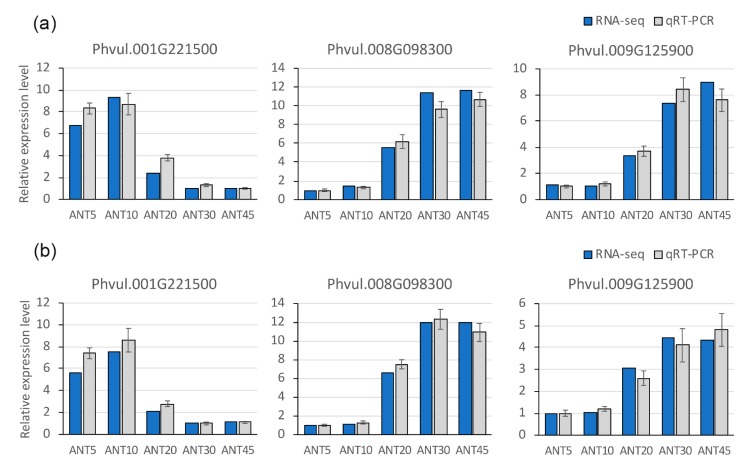
Validation of gene expression obtained from RNA-seq by qRT-PCR. The dynamic expression patterns of Phvul.001G221500, Phvul.008G098300, and Phvul.009G125900 transcriptions factors evaluated by qRT-PCR in PHA-1037 (**a**) and PMB0255 (**b**) accessions. Error bars represent the standard deviation of qRT-PCR from three biological and two technical replicates.

To provide insights into the transcriptional regulatory activity that is essentially attributable to the pod maturation process, both up- and down-regulated differentially expressed fruit maturation genes shared between common bean and Arabidopsis were used to perform a transcription factor binding site (TFBS) enrichment analysis. This analysis allowed for the identification of three transcription factors whose TFBSs were significantly over-represented in the promoter regions of candidate maturation genes (Table S7). A total of 117 candidate maturation genes were identified as targets of these three transcription factors (Table S7), ranging from 24 to 63 for Phvul.009G125900 and Phvul.001G221500, respectively. Among them, both up- and down-regulated genes were found as targets of these three transcription factors, suggesting that they may act as transcriptional activators or repressors. Therefore, the transcription factors identified are more likely to be of major biological relevance during the pod maturation process. One of them, Phvul.001G221500, was down-regulated at maturation stages and encodes an MYB transcription factor homologous to the Arabidopsis *MYB61* gene, which has been previously involved in both cell wall synthesis and regulation of plant resource allocation [48,49,50]. The other two, Phvul.008G098300 and Phvul.009G125900, were up-regulated in mature pods and code for homeodomain-like and NAC transcription factors, respectively. The function of the Arabidopsis homolog of Phvul.008G098300 has not been described so far, whereas Phvul.009G125900 is homologous to the Arabidopsis *ATAF1* gene. Notably, *ATAF1* has been recently identified to act as a core transcriptional activator of senescence by directly regulating both chloroplast maintenance and senescence-related signaling cascades [43]. To validate the accuracy of expression patterns obtained for these three transcription factors, their relative expression levels were experimentally assessed using quantitative real-time PCR (qRT-PCR) and independent biological samples. The qRT-PCR analysis showed that transcript abundance of Phvul.001G221500 was higher in immature pods, whereas Phvul.008G098300 and Phvul.009G125900 transcription levels were increased at mature stages. Thus, the relative expression levels obtained from qRT-PCR were consistent with the normalized read counts from RNA-seq in both genotypes at all developmental stages here analyzed (Figure 6), proving the reliability of the RNA-seq data.

## 3. Discussion

### 3.1. Transcriptome Analyses Reveal Pod Maturation Specific Genes of Common Bean

In-depth information about gene expression is crucial to understanding the molecular mechanisms underlying fruit maturation, mainly in legume crop species like a common bean in which this process determines breeding goals like yield and fruit quality. In this study, a comprehensive transcriptome analysis of pod development has been performed in two contrasting accessions of common bean with the aim to reveal those gene expression changes that, independently of the genotype diversity, are involved in the maturation process of common bean. In both accessions, through a multivariate analysis of gene expression, ANT5 and ANT10 were confirmed as immature stages and ANT20, ANT30, and ANT45 as mature stages, in accordance with phenotypic data (see Figure 1) and other previously reported studies [33]. A comparative gene expression analysis showed that 2487 DEGs were shared by PMB0225 and PHA1037 accessions (Tables S2.1 and S2.2), indicating that a relatively large amount of the transcriptional program operating during maturation is shared between both cultivars. Indeed, regulation of transcription and transcription factor activity appeared as an important biological process and molecular function categories, respectively (see Figure 4). As regards to the transcriptional control, several genes of the MAPK signaling pathway also modify their transcript accumulation level during pod maturation, which confirms the essential role that phosphorylation plays as a regulatory mechanism of gene expression [51,52].

Enrichment analysis in KEGG pathways revealed that 165 of the 2487 DEGs shared by both accessions participate in essential physiological processes required for fruit maturation in common bean and other related species [5,40]. Furthermore, the comparative analysis of pod maturation in common bean and Arabidopsis has highlighted that 710 of the common bean DEGs are homologous to Arabidopsis genes already described as implicated in the maturation process [41], which also display similar gene expression patterns (i.e., genes up-/down-regulated in one species are also up-/down-regulated in the other). Several members of NAC, GRAS, and WRKY transcription factor families deserve special mention, which have been reported to play significant roles in regulating fruit maturation-associated and dehiscence processes [42,43,44,45,46]. Together, these transcriptome features have allowed for the identification of 781 DEGs (235 up- and 546 down-regulated genes, once redundancy between genes listed in Tables S4 and S5 was eliminated) that should be considered as specifically involved in pod maturation, and represent the first maturation gene set reported in common bean, so far.

Furthermore, results from TFBS enrichment analysis allowed for the identification of key components of the transcriptional regulatory activity underlying pod maturation. Among them, Phvul.001G221500 and Phvul.009G125900, the homologs of the Arabidopsis *MYB61* and *ATAF1* genes, showed opposite expression patterns (i.e., *MYB61* and *ATAF1* down- and up-regulated at maturation stages) suggesting a different functional role between these two transcription factors during the pod maturation process. In Arabidopsis, *MYB61* participates in conditioning plant resource allocation by regulating cell wall synthesis genes [48,49]. This function is conserved in rice, since it has been described that *OsMYB61a* can bind to the promoters of secondary wall cellulose synthase genes regulating their expression [53,54]. The Phvul.001G221500 transcription factor is highly expressed in sink tissues such as developing pods (Table S2). Likewise, genes associated with cell wall metabolism, such as those encoding beta-glucosidase (Phvul.001G076700) or pectin methylesterase inhibitor (Phvul.009G224200 and Phvul.009G053000) enzymes, were found among its targets (Table S7), suggesting a putative role for Phvul.001G221500 in the regulation of resource allocation. In regards to the Arabidopsis *ATAF1* gene, it promotes senescence by regulating chloroplast maintenance and senescence transcriptional cascades [43]. In keeping with its putative role as a senescence promoter, Phvul.009G125900 expression was found to be up-regulated at mature pod stages (Table S2). It is further interesting to note that TFBSs for Phvul.009G125900 were identified in genes associated with chloroplast development and photosynthesis, as well as senescence regulatory networks (Table S7). Among them, Phvul.006G178400, a gene coding for a subunit of magnesium chelatase required for chlorophyll biosynthesis [55], and Phvul.008G288500 which encodes a protein kinase that is homologous to the Arabidopsis *SAI1* gene, whose loss of function mutations affect the oxidative stress responses and iron distribution within chloroplasts [56]. Furthermore, it was found TFBSs for Phvul.009G125900 among DEGs encoding cell-death-related proteins such as cathepsin B (Phvul.001G220100) and cysteine (Phvul.002G049500 and Phvul.009G170800) proteases, which supports that fruit maturation involves an apoptotic program [41].

### 3.2. Pod Maturation Is Regulated at Translational Level and Involves Important Expression Changes of Photosynthesis-Related Genes

Together with the transcriptional regulation mentioned above, this study’s results indicated a translational control of pod maturation in common bean. Indeed, RNA-seq analysis showed that, when pod maturation is initiated and the maturation process proceeds, there is a marked decrease in abundance of the proteins involved in the ribosome assembly and translation machinery. Although the KEGG enrichment analysis of 2487 candidate maturation genes did not show this genetic pathway as being significant, it appears as significantly enriched when maturation genes shared by common bean and *A. thaliana* were used for the enrichment test (Tables S5 and S6).

During pod maturation, both gross photosynthesis and dark respiration decreased; consequently, the photosynthetic machinery is progressively dismantled and chlorophyll content is lost [8,9,10]. Outstanding results of the RNA-seq analysis here reported indicate that photosynthesis and photosynthesis–antenna protein-coding genes were down-regulated at pod mature stages (Figure 5b, Table S4.2), thus likely causing a decrease in chlorophyll content of pods. Interestingly, these pathways were also enriched in the down-regulated maturation genes shared with Arabidopsis (Table S6). Chlorophylls are accumulated in tissues where chlorophyll-binding proteins of the photosystem I (PSI) and II (PSII) are produced [57]. Accordingly, reduced levels of transcripts of genes coding for chlorophyll-binding proteins have been detected between unripe and ripe stages of tomato fruits [58,59]. Likewise, a loss of chlorophyll and photosynthetic activity (PSI and PSII) was observed during the maturation program in seeds and siliques of Brassicas [60]. In agreement with all these previous observations, genes coding for proteins involved in the PSI (PsaD, PsaE, PsaH, PsaK, and PsaL) and PSII (PsbO, PsbP, PsbQ, PsbS, and PsbY), as well as photosynthetic electron transport (PetE and PetF) were down-regulated at mature stages of common bean pods (Figure 5b, Table S4.2) coinciding with the loss of their green color (see Figure 1). All these results corroborate that the significant reduction of photosynthesis, as part of the senescence developmental program, is a key step for pod maturation in common bean.

Furthermore, a relationship between photosynthesis and ribosomal machinery has been previously reported in Arabidopsis [61]. Thus, the decreased abundance of chloroplast 16S rRNA promoted by the lack of ribosomal protein S5 leads to the suppression of a great number of core components of PSI and PSII [61]. In summary, according to this study’s findings, the pod maturation process in common bean leads to specific transcriptome changes that accelerate the breakdown of photosynthetic and ribosomal machinery. Molecular breeding should take the extent of these DEGs into account to develop strategies for modulating pod maturation and, hence, to improve yield and pod quality of common bean crops.

### 3.3. Genes Regulating Metabolism Pathways Modify Their Expression Pattern during Pod Maturation

Physiological, genetic, and molecular changes that occur during fruit maturation lead to modifications in the primary and secondary metabolism in both fleshy- and dry-fruited species [1,2,3,4,5,6]. Along this developmental process, chlorophyll degradation is accompanied by biosynthesis of carotenoids and anthocyanins, which determine changes in fruit color during ripening [9]. In common bean and soybean, the green color of immature pods is largely due to the chlorophylls, whose concentration decreases gradually with pod maturation [62,63]. It was found that genes coding for proteins related to the porphyrin and chlorophyll metabolism were down-regulated at mature pod stages (Table S4.2), indicating that these genes may play an important role in determining chlorophyll content during pod maturation in common bean. Among them, the homologous to the Arabidopsis *STAY-GREEN* (*SGR*, Phvul.009G132100), which encodes a magnesium-dechelatase that has been described as being involved in the chlorophyll degradation pathway [10,64]. SGR homologs have been identified as those responsible for stay-green phenotypes in tomato, pepper, rice, and Arabidopsis, in which chlorophyll degradation normally occurs at the onset of fruit ripening [65,66,67]. In addition, the tomato SGR regulates lycopene and β-carotene biosynthesis during fruit ripening [68]. Similarly, transcriptome analyses described in this work clearly show a significant up-regulation of several genes involved in carotenoid biosynthesis at pod maturation stages, such as those encoding beta-carotene 3-hydroxylase (Phvul.007G074200), 9-cis-epoxycarotenoid dioxygenase (Phvul.005G051600), and abscisate beta-glucosyltransferase (Phvul.003G047200) (Table S2.1), indicating that carotenoid biosynthesis and chlorophyll degradation are coordinated metabolic pathways essential for pod maturation in common bean.

Chlorophyll degradation is also regulated by jasmonate [12,13], which in turn is synthesized from alpha-linolenic acid [14,15]. Hence, it might be expected that both compounds would have the same effect to stimulate the loss of chlorophylls [16]. In agreement with this hypothesis, it was found that several genes integrating the alpha-linolenic acid metabolism pathway are up-regulated during common bean maturation (Figure 5a, Table S4.1), among them, the homologs to the Arabidopsis *ACYL-COA OXIDASE* (Phvul.002G056700, Phvul.010G076000), *LIPOXYGENASE3* (Phvul.010G128800), and *12-OXOPHYTO, DIENOIC ACID REDUCTASE* (Phvul.001G000800, Phvul.001G096300, Phvul.003G131500, Phvul.003G131600), all of them previously described as implicated in regulating the biosynthesis of jasmonate from alpha-linolenic acid [14,15]. Such results indicate that jasmonate-mediated chlorophyll metabolism should be conserved in dry-fruited species as Arabidopsis and common bean, and that the aforementioned genes should be used as molecular tools for the breeding of fruit quality in the latter one.

The RNA-seq analysis has also revealed that common bean pods showed reduced expression levels of genes involved in the fatty acid biosynthesis pathway during the maturation process (Table S4.2). Thus, genes coding for enzymes of this pathway, i.e., acetyl-CoA carboxylase 1 (Phvul.004G034300, Phvul.004G035000, 3-oxoacyl-synthase II (Phvul.002G300400), enoyl-reductase (Phvul.008G134300), fatty acyl-ACP thioesterase B (Phvul.009G200200), and long-chain acyl-CoA synthetase (Phvul.003G010600, Phvul.009G124800) enzymes, were down-regulated at pod mature stages. These observations are consistent with the wax accumulation on the pod surface observed at the onset of maturation, associated with a decrease in expression of genes coding for enzymes that modify cutin fatty acids [40].

Previous research has shown an increase in the antioxidant level during fruit maturation in response to the enhanced accumulation of reactive oxygen species associated with senescence [69,70]. In this regard, arachidonic acid metabolism pathway plays an important biological role in preventing oxidative damage [71]; indeed, two enzyme activities involved in the arachidonic acid pathway, superoxide dismutase and glutathione peroxidase (GPX), were induced during snap pea (*Pisum sativum*) pod maturation process [72]. In agreement, an increased expression of two genes of the same pathway, the homologs to Arabidopsis *GPX6* (Phvul.002G288700) and *GPX8* (Phvul.002G288800), were found at pod maturation stages in this study (Table S4.1), suggesting that arachidonic acid probably helps to protect common bean pods from oxidative stress.

## 4. Materials and Methods

### 4.1. Plant Material and Sample Collection

Common bean cultivated breeding line PMB0225 and the weedy PHA1037 accessions (Figure 1) from the Andean gene pool were grown in a growth chamber (20–25 °C, relative humidity 70%–90%, 8/16 h light/dark regime). PMB0225 is a Spanish improved line adapted to long-day photoperiod, which shows indeterminate erect growth habit type II, white flowers, indehiscent and stringless pods, as well as large white seeds. PHA1037 is a long-day photoperiod-sensitive nuña popbean germplasm accession from Bolivia that possesses an indeterminate climbing growth habit type IV and has purple flowers, dehiscent, and stringy pods together with large red seeds.

Ninety flowers on 18 common bean plants per genotype were tagged during flowering. Five pod stages (exponential growth-green, linear growth-green, cessation of pod elongation-green, maturation-green, and maturation-yellow) were numbered from ANT5 to ANT45, and collected at 5, 10, 20, 30, and 45 days after anthesis (DAA), respectively. Six uniformly sized pods were sampled at every stage (one biological replicate). A total of three biological replicates were collected for each genotype, at developmental stages ANT5 (R3-I), ANT10 (R3-II), ANT20 (R5-III), ANT30 (R5-IV), and ANT45 (R7-IV). Seeds were manually removed (except at ANT5) to exclude any embryo contribution to the data set, with the aim to increase the probability of identifying genes involved specifically in the pod maturation process. After removing the seeds, the pod was cut into 0.8–1.0 cm^3^ pieces from pedicel, peduncle, and medium, and stored in liquid nitrogen.

### 4.2. RNA Extraction, Library Construction and RNA-Seq

Total RNA was extracted using TriFast Reagent (Peqlab, Erlangen, Germany) according to the manufacturer’s instructions. The RNA sample quality was assessed by NanoDrop 2000C (Thermo Scientific, Wilmington, USA), agarose gel electrophoresis, and Agilent 2100 BioAnalyzer (Agilent Technologies, Santa Clara, CA, USA). Only the RNA samples with 1.8/2.1 (260/280) and 2.0/2.5 (260/230) ratios and a RIN (RNA integrity number) value >7.0 were sequenced. Sequencing libraries were generated using the NEBNext Ultra Directional RNA Library Prep Kit for Illumina (New England Biolabs, Ipswich, MA, USA). The cDNA library insert sizes ranged from 250 to 300 bp. The complete libraries were purified with AMPure XP system (Beckman Coulter, Beverly, MA, USA) and qualified by Agilent Bioanalyzer 2100 system (Agilent Technologies, Santa Clara, CA, USA). Finally, libraries were sequenced on an Illumina Hiseq 2500 platform (Illumina Inc., San Diego, CA, USA), and 150 bp paired-end reads were generated. Raw sequencing reads were deposited at NCBI Short Read Archive (SRA) under the BioProject accession code PRJNA578479 [73].

### 4.3. RNA-Seq Bioinformatics Protocol

The diagram shown in Figure 2 describes the RNA-seq pipeline used for read pre-processing, genome alignment, transcriptional level quantification and differential expression analysis. Quality control and adapter trimming of paired-end short reads were carried out by means of FastQC [74] and Trim-Galore [75] by using its default parameters. All samples show high read coverage, percentages of correct mapped reads being always above 90% (Table S1). The filtered paired-end short reads were aligned to the reference genome (*Phaseolus vulgaris* v2.1) by means of STAR [76] with default options. The Rsamtools [77], GenomicFeatures, GenomicAlignments [78], and SystemPipeR [79], Bioconductor R packages were used for transcript assembly and abundance estimation. To check the consistency of RNA-seq replicates, the common top-ranked 100 expressed genes among samples were selected in all replicates for hierarchical clustering analysis based on log(FPKM+1) values. Hierarchical clustering was conducted using *heatmap.2* and *hclust* functions (distance method = “maximum” and cluster method = “average”) from the *gplots* R package [80]. In some of the samples, one of the replicates showed a clustering behavior disagreeing with the other two replicates in the sample, thus meaning that the discarded replicate clustered with other samples than its own, and was therefore discarded for further analyses (Figure S1, Table S1).

### 4.4. Determination of Differential Gene Expression

**Figure 7:**
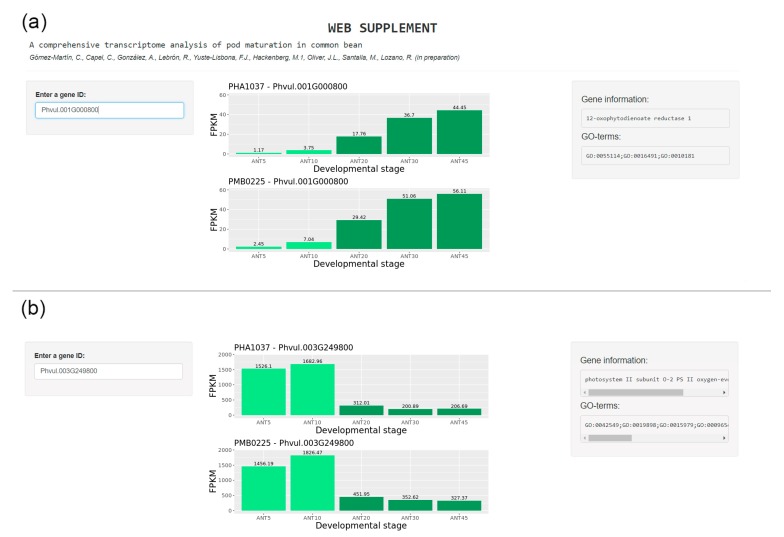
Working example of the Shiny webserver page showing the expression profiles of (**a**) up-regulated gene Phvul.001G000800 (12-oxophytodienoate reductase 1) and (**b**) down-regulated gene Phvul.003G249800 (photosystem II subunit O-2 PS II oxygen-evolving complex 1).

DEGs along the pod maturation process were identified by means of custom python scripts [36]. In these scripts, a fold change (FC) was computed in all pairwise comparisons between mature and immature stages. Then, a |FC|>2 between mature and immature stages in all pairwise possible comparisons was required to consider a differentially expressed gene (up or down depending on the sign of the FC). A Shiny Web App [81] was implemented to visualize the expression profiles of the identified maturation genes [37]. In Figure 7, an example of this web tool is shown. To this end, two genes were chosen, one up-regulated and one down-regulated. The up-regulated one is Phvul.001G000800 (12-oxophytodienoate reductase 1), which is part of the alpha-linoleic acid metabolism pathway. The down-regulated gene shown is Phvul.003G249800 (photosystem II subunit O-2 PS II oxygen-evolving complex 1), which is part of the photosynthesis pathway.

### 4.5. Multivariate Analysis

A correspondence analysis [34,35] was carried out using the FactoMineR and factoextra R packages. Venn diagrams were made using the VennDiagram R package [82]. Heat maps and hierarchical clustering were obtained with the Morpheus tool [83].

### 4.6. Functional Annotation and Pathway Enrichment

GO-term enrichment analyses were performed using AgriGO V2 [84] and reduced using REVIGO [85]. Enriched GO terms are represented as scatterplots after a redundancy reduction that summarizes GO terms’ semantic similarities. In these representations semantically similar GO terms remain close together in the plot, although the semantic space units have no intrinsic meaning.

KEGG pathway enrichment [38] was carried out for up-regulated and down-regulated genes independently using enrichKEGG and dotplot functions of the clusterProfiler package [86] and an R custom script [87]. Enriched pathways were plotted using Pathview [39] and an R custom script [87].

### 4.7. Comparative Analysis of Common Bean and Arabidopsis Fruit Maturation Genes

Candidate maturation DEGs were compared with those obtained by Mizzotti et al. [41] in Arabidopsis. First, a list of common bean homologous genes to those found in Mizzotti et al. [41] was obtained using Phytomine [88]. Then, maturation candidate genes were compared with the resulting list and only the shared genes by both lists were selected. Lastly, a KEGG enrichment analysis (described above) was computed.

### 4.8. Enrichment Analysis of Transcription Factor Binding Sites (TFBSs)

The TFBS enrichment analysis was carried out using the plant regulatory data and analysis platform PlantRegMap [89], which contains 2177 transcription factors (1867 loci) classified into 58 families and their respective targets for *P. vulgaris* [89]. Initially, TFBSs in the promoter region (transcription start site + 500 bp – ~ 100 bp) of common bean genes were detected using three methods: (i) motif: FIMO [90] was used to identify the high-quality binding motifs of a transcription factor with threshold 1 × 10^–5^, and a potential interaction was assigned if there was at least one binding site in the promoter of the gene; (ii) motif + conserved elements (motif_CE): an interaction was assigned only if more than 50% length of a binding site overlaps to conserved elements (identified by RPHAST [91]; and (iii) FunTFBS: an interaction was assigned only if there was at least one functional binding site (identified by the FunTFBS method [89]) in the promoter of the gene. Afterwards, common bean genes homologous to those from Arabidopsis reported by Mizzotti et al. [41] were used to perform a TFBS enrichment analysis. The identified TFBSs in promoter sequences of fruit maturation genes shared between common bean and Arabidopsis were compared against total TFBSs in promoters of all common bean genes. Finally, Fisher’s exact test was performed to find significant (*P* ≤ 0.05) TFBS enrichment in candidate maturation genes.

### 4.9. Quantitative Real-Time PCR (qRT-PCR)

To validate the findings of the RNA-seq analysis, RNA isolated from each common bean genotype (at fruit developmental stages ANT5, ANT10, ANT20, ANT30, and ANT45) was used to construct a cDNA library. The cDNA was synthesized from 100 ng of total RNA by M-MuLV reverse transcriptase (Fermentas Life Sciences, Hanover, MD, USA) with a mixture of random hexamer and oligo(dT)18 primers. SYBR Green PCR Master Mix kit (Applied Biosystems) was used to perform qRT-PCR reactions in a 7300 Real-Time PCR System (Applied Biosystems, Foster City, CA, USA), according to the manufacturer’s instructions. A standard curve containing 5 points was made using 1:10 serial dilutions of stock cDNA to empirically determine the primer efficiency value for each primer pair, which ranged from 89.8% to 95.2% for Phvul.008G098300 and Phvul.001G221500 gene, respectively. All reactions were performed in duplicate at a volume of 10 µL, containing 1 µL of cDNA and 300 nM of each specific primer. The conditions for the qRT-PCR amplifications were as follows: 95 °C for 10 min, followed by 40 cycles at 95 °C for 15 s and 60 °C for 1 min. At the end of each reaction, a melting curve analysis of amplification products was performed to confirm that only one PCR product was amplified and detected. Results were processed using the ΔΔCt calculation method [92], expressed in arbitrary units and normalized by comparison to the housekeeping *UBIQUITIN* gene (Phvul.001G193800). Specific primer pairs for Phvul.001G193800 (forward 5′- TTACATGCGCTCTTGGACTG - 3′ and reverse 5′- CGAACACTTGGAGGCTTTTC - 3′), Phvul.001G221500 (forward 5′- CCAAAACTAGCTGGCCTTCA - 3′ and reverse 5′- ACGCCCCTCTCTTCAAATCT - 3′), Phvul.008G098300 (forward 5′- AACCTCGTTACCCGCTATAAGG - 3′ and reverse 5′ - GCTTCTGCCCACAACATTCT - 3′), and Phvul.009G125900 (forward 5′- GAACAGCCTAAGGTTGGATGAT - 3′ and reverse 5′ -TCACAACTACGTCGGTGCTC - 3′) were used to perform qRT-PCR experiments.

## 5. Conclusions

Pod growth and maturation are essential developmental processes not only for their biological and evolutionary significance in seed development and dispersal, but also for their implications on determining yield quality and nutritional value. Despite its significant implications in breeding, the expression pattern of key genes involved in regulating pod growth and maturation in this legume species has remained largely unknown so far. The set of analyses performed in this study provides transcriptional insights into the key genes and pathway signatures relevant to pod maturation, supplying not only potentially useful information for common bean breeding programs, but also opening up new opportunities for future research on pod growth and maturation.

## Supplementary Materials

The following are available online at https://www.mdpi.com/2223-7747/9/4/545/s1, Figure S1: Hierarchical clustering of the common top-ranked 100 expressed genes, which was obtained from all replicates and samples in both PMB0225 and PHA1037 cultivars. Table S1: Quality and read mapping report. Table S2.1 and S2.1: FPKM values in up-regulated and down-regulated genes in stages ANT20, ANT30 and ANT45 (mature stages) in comparison to stages ANT5 and ANT10 (immature stages). Table S3.1 and S3.2: Enriched GO terms in up-regulated and down-regulated candidate maturation genes. Table S4.1 and S4.2: Up-regulated and down-regulated specific maturation genes grouped by KEGG pathways. Tables S5.1 and S5.2: Homologous up-regulated and down-regulated maturation genes shared by *Arabidopsis thaliana* and *Phaseolus vulgaris*. Table S6: KEGG pathway enrichment in fruit maturation genes shared by *Arabidopsis thaliana* and *Phaseolus vulgaris*. Table S7: Transcription factors whose TFBSs were significantly over-represented in the promoter regions of fruit maturation genes shared between common bean.
