# Supplementary material for: Transcriptional Dynamics and Candidate Genes Involved in Pod Maturation of Common Bean (Phaseolus vulgaris L.)
Source: Plants (Basel). 2020 Apr 22;9(4):545. doi: 10.3390/plants9040545 (PMC7238275; doi:10.3390/plants9040545)
Supplement: Supplementary file 1 [file plants-09-00545-s001.zip › SupplementaryFiles/FigureS1.pdf]

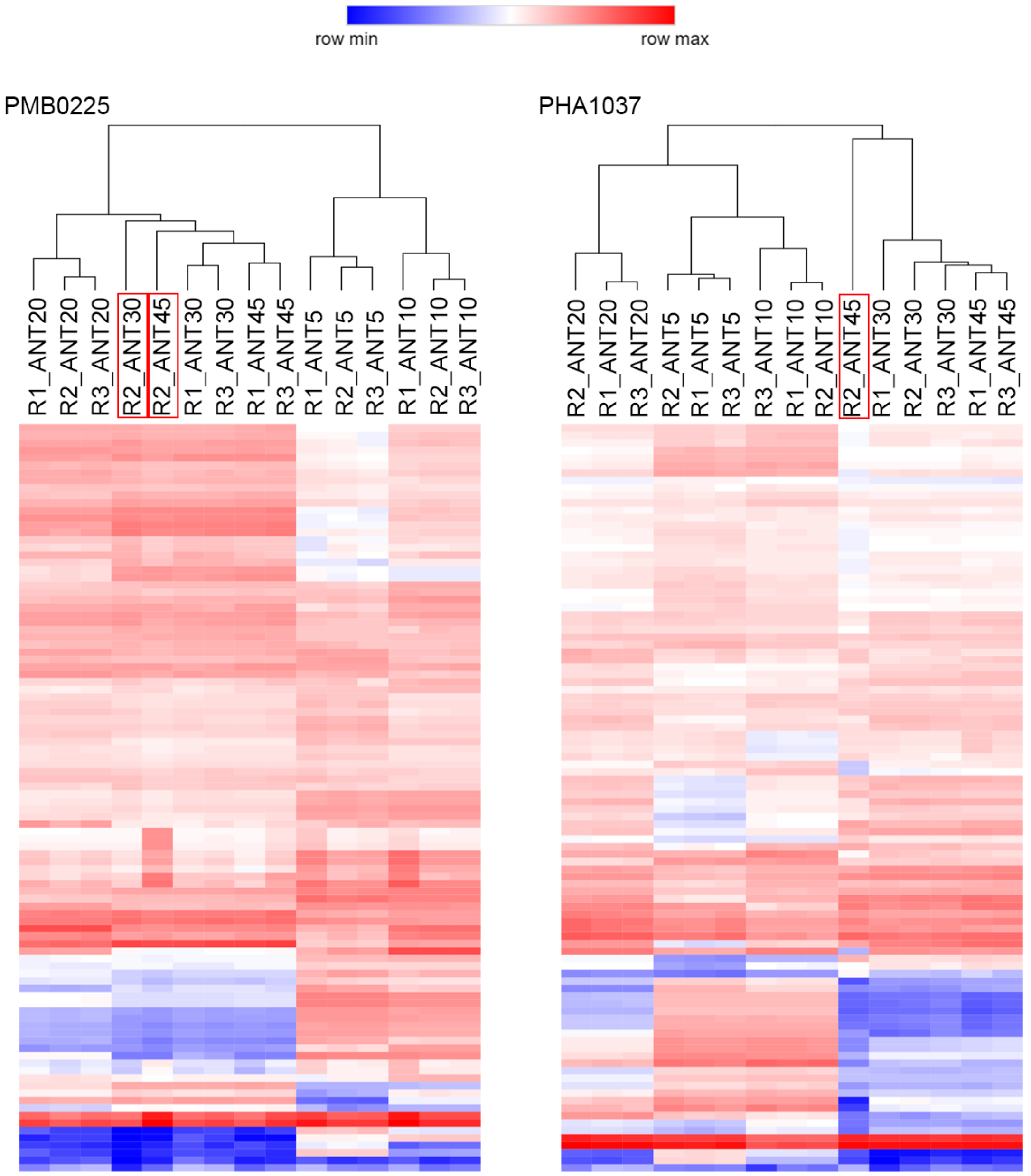

Figure S1. Hierarchical clustering of the common top-ranked 100 expressed genes, which was obtained from all replicates and samples in both PMB0225 and PHA1037 cultivars. The *heatmap.2* and *hclust* functions (distance method = 'maximum' and cluster method = 'average') from the *gplots* R package (https://cran.r-project.org/package=gplots) were used for clustering analysis based on log(FPKM+1) values. The replicates framed in red showed a clustering behavior discordant to the other two replicates for the same developmental stage, and therefore they were discarded for further analyses.
